# Smooth and accurate predictions of joint contact force time-series in gait using over parameterised deep neural networks

**DOI:** 10.3389/fbioe.2023.1208711

**Published:** 2023-07-03

**Authors:** Bernard X. W. Liew, David Rügamer, Qichang Mei, Zainab Altai, Xuqi Zhu, Xiaojun Zhai, Nelson Cortes

**Affiliations:** ^1^ School of Sport, Rehabilitation, and Exercise Sciences, University of Essex, Colchester, United Kingdom; ^2^ Department of Statistics, Ludwig-Maximilians-Universität München, Munich, Germany; ^3^ Munich Center for Machine Learning, Munich, Germany; ^4^ Faculty of Sports Science, Ningbo University, Ningbo, China; ^5^ Research Academy of Grand Health, Ningbo University, Ningbo, China; ^6^ Auckland Bioengineering Institute, The University of Auckland, Auckland, New Zealand; ^7^ School of Computer Science and Electrical Engineering, University of Essex, Colchester, United Kingdom; ^8^ Department of Bioengineering, George Mason University, Fairfax, VA, United States

**Keywords:** locomotion, running biomechanics, walking biomechanics, musculoskeletal modelling, deep learning, machine learning

## Abstract

Alterations in joint contact forces (JCFs) are thought to be important mechanisms for the onset and progression of many musculoskeletal and orthopaedic pain disorders. Computational approaches to JCFs assessment represent the only non-invasive means of estimating *in-vivo* forces; but this cannot be undertaken in free-living environments. Here, we used deep neural networks to train models to predict JCFs, using only joint angles as predictors. Our neural network models were generally able to predict JCFs with errors within published minimal detectable change values. The errors ranged from the lowest value of 0.03 bodyweight (BW) (ankle medial-lateral JCF in walking) to a maximum of 0.65BW (knee VT JCF in running). Interestingly, we also found that over parametrised neural networks by training on longer epochs (>100) resulted in better and smoother waveform predictions. Our methods for predicting JCFs using only joint kinematics hold a lot of promise in allowing clinicians and coaches to continuously monitor tissue loading in free-living environments.

## 1 Introduction

*In-vivo* measurement of joint contact forces (JCF) provides highly accurate measures of tissue loading (Bergmann et al., 2016). Tissue loading information is crucial for the understanding of disease progression, injury prevention, rehabilitation, and even the designing of new artificial joints or limbs. However, *in-vivo* measurements require invasive instrumentation (Bergmann et al., 2016), which cannot be readily extended to the study of both healthy and pathological participants. To circumvent the problems of *in-vivo* measurements, computational musculoskeletal models have been developed, which provide a non-invasive method of estimating JCFs (Delp et al., 2007). To calculate JCFs, musculoskeletal modelling software require two sources of information–body segment kinematics optical cameras, and ground reaction forces (GRFs) from force plates. Logistically, it is very challenging to acquire GRFs outside a laboratory, given that force plates have to be either embedded into the ground or integrated within a bulky instrumented treadmill.

Increasingly, researchers have turned to machine learning (ML) to train a statistical model which learns complex patterns that map easier-to-collect biomechanical predictors onto harder-to-collect biomechanical outcomes in the laboratory (Liu et al., 2009; Johnson et al., 2019b; Stetter et al., 2020; Liew et al., 2021; Boukhennoufa et al., 2022). Current studies have used predictors such as body segment kinematics from optical cameras (Johnson et al., 2019a; Giarmatzis et al., 2020; Liew et al., 2021; Boukhennoufa et al., 2022), inertial measurement units (IMUs) (Lee and Park, 2020; Mundt et al., 2020; Stetter et al., 2020; Wang et al., 2020), markerless motion capture (Boswell et al., 2021), or using a combination of wearable sensors like IMUs, electromyography (EMG), and pressure insoles (He et al., 2019; Rane et al., 2019; Zhu et al., 2020; Camargo et al., 2022; Moghadam et al., 2023). Most ML studies in biomechanics have focused on the prediction of GRFs and joint moments during various locomotion patterns, such as walking and side-step cutting (Liu et al., 2009; Johnson et al., 2019b; Stetter et al., 2020; Wang et al., 2020). Neural networks are the most common ML method used in contemporary biomechanics research for predicting kinetic variables (Johnson et al., 2019a; Johnson et al., 2019b; He et al., 2019; Stetter et al., 2020; Wang et al., 2020; Boswell et al., 2021; Boukhennoufa et al., 2022), although methods such as gradient boosting have been used (Wang et al., 2020; Camargo et al., 2022).

Although predicting GRFs and joint moments have their clinical utility, such measures may not accurately reflect tissue load measures, such as JCFs (Walter et al., 2010; Matijevich et al., 2019). The capacity to accurately estimate JCFs using ML may radically transform the way we measure the biomechanical markers of joint diseases and monitor the effects of treatments. Increasingly, studies have begun exploring the role of ML in estimating muscle forces (Rane et al., 2019; Moghadam et al., 2023) and JCFs (Ardestani et al., 2014; Rane et al., 2019; Giarmatzis et al., 2020; Zhu et al., 2020), by training ML models on outcomes derived from force-sensor implants (Ardestani et al., 2014; Zhu et al., 2020), or from musculoskeletal models (Rane et al., 2019; Giarmatzis et al., 2020; Moghadam et al., 2023). For JCFs, only the knee has been the focus of investigation (Ardestani et al., 2014; Rane et al., 2019; Giarmatzis et al., 2020; Zhu et al., 2020), and only walking has been studied (Ardestani et al., 2014; Rane et al., 2019; Giarmatzis et al., 2020; Zhu et al., 2020).

Although an increasing amount of research has been done in developing ML models for predicting joint kinetics, several methodological issues remain. If ML models learn statistical relationships between a set of inputs and output, it is interesting to speculate as to the nature of the relationships learned, and the transferability of such knowledge across different settings. At its core, the relationship that connects motion to forces is determined by Newton’s Laws of Motion. If the relationship learned by ML models are approximating known physical laws, it suggests that ML models can be applied to biomechanical domains beyond that trained by the model. For example, the ability of ML models trained during a walking task, but applied to a running task, and *vice versa*. A related issue that affects the transferability of ML performance is the number of epochs that neural networks are trained on (or the number of iterations when using boosting). In the wider ML fields, early stopping of the training process when a prior criterion threshold is reached is thought to reduce statistical overfitting and improve the transferability of performance–i.e. the Bias-Variance tradeoff. However, ML models in biomechanics have used a fixed number of training epochs/iterations, without evaluating if this affect prediction performance.

This study aims to develop ML models to predict the outcomes of lower-limb JCFs of the ankle, knee, and hip joints, using 3D joint angles obtained using optical cameras. We hypothesise that the prediction errors of our ML models would be less than current minimal detectable change (MDC) values of traditional musculoskeletal modelling practices (e.g., range between 0.43 and 1.53 bodyweight [BW] (Price et al., 2017). The secondary aim of the present study is to determine the effect of varying training epochs on ML performance, with the hypothesis that a greater number of training epochs will always lead to a drop in prediction performance. The third aim of this study is to determine if training an ML model on data from one gait type can predict outcomes from another gait type. We hypothesised that if ML models are learning statistical relationships that reflect fundamental laws of physics, then the performances of ML models trained and tested on different gait types will be similar to the performances of models trained and tested on identical gait types. The last aim is to determine if increasing the sample size by combining both walking and running datasets can improve the prediction performance, compared to ML models trained only on a single gait dataset.

Findings from this study represent several innovation points. First, we will be able to determine if ML can be used to predict JCFs across the three major lower-limb joints in both light and high-impact activities. Second, we will be able to determine the impact of over parameterisation in deep neural networks on the smoothness of predictions of our JCFs. Lastly, we will be able to determine if ML models can be translated across gait patterns, providing insights into the nature of relationships learnt by ML in biomechanics.

## 2 Methods

### 2.1 Design

**FIGURE 1:**
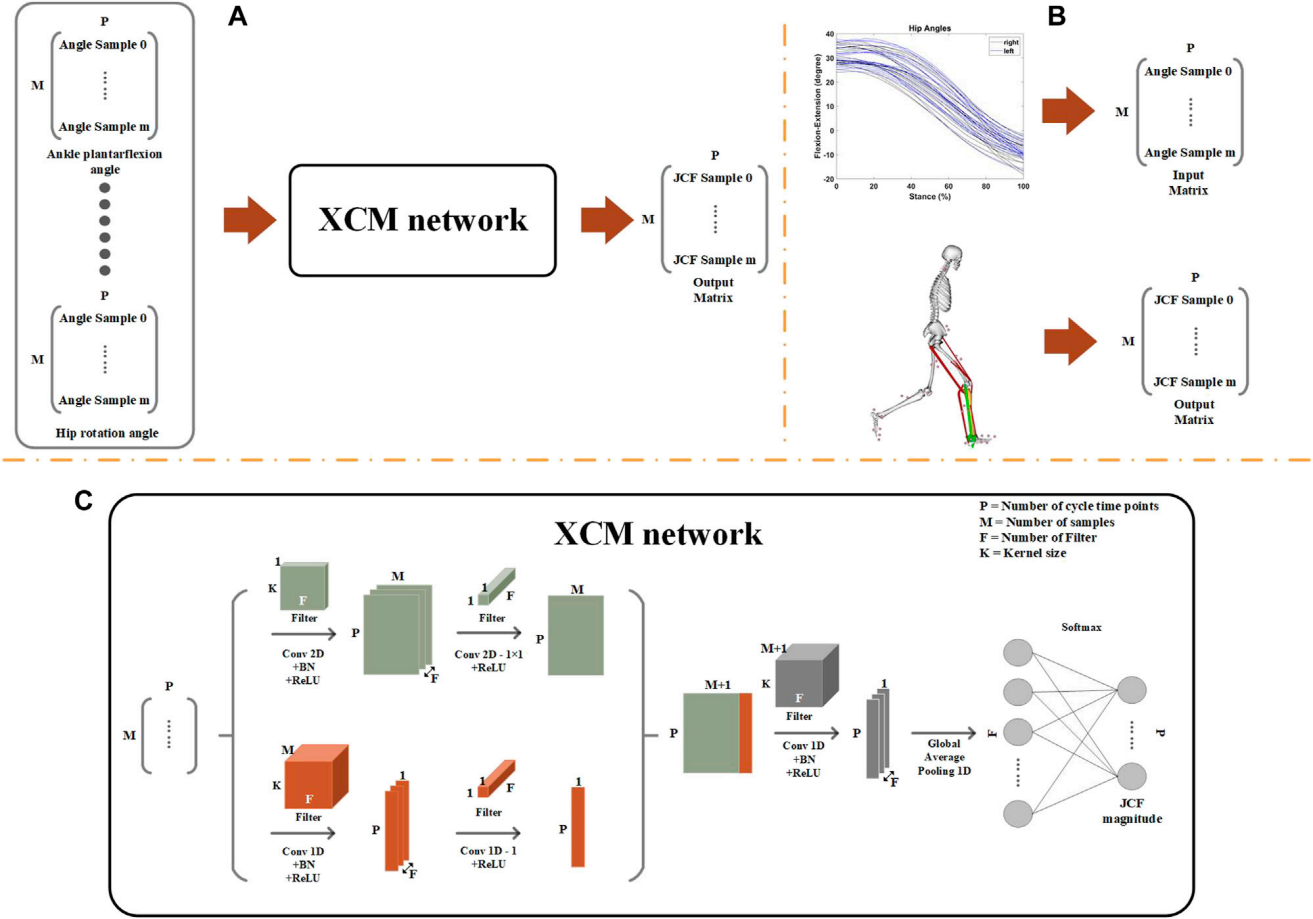
**(A)** General workflow of the deep learning modelling approach, with the three-dimensional joint kinematics used as the predictors, and joint contact forces as outcomes; **(B)** Data organisation of the multivariate time-series predictors and univariate outcomes; and **(C)** High-level overview of the XCM model architecture.

This is a secondary data analysis of a musculoskeletal modelling study of walking and running, the data of which, is publicly available (Mei et al., 2022). Herein, we summarise the experimental procedure used to collect the data, with specific details provided in the main article (Mei et al., 2022). Figure 1 represents a workflow of the methods employed in this study.

Walking and running biomechanics assessment was performed on 20 recreational runners (20 males, mean (one standard deviation [SD] age: 25.8 (1.6) years, height: 1.73 (0.05) m, and mass: 67.8 (5.3) kg)). Walking and running assessments were conducted along a 20 m runway, with marker trajectories collected using an eight-camera optical camera system (200 Hz, Vicon Metrics Ltd., Oxford, UK)), and an inground force platform (1000 Hz, AMTI, Watertown, MA, United States) in a motion capture laboratory. Anatomical and technical markers were placed on the trunk, pelvis, bilateral thighs, shanks, and feet, to create an eight-segment model.

### 2.2 Protocol

Participants performed two sets of biomechanics assessments, before and after a 5 km treadmill run. For each set of assessments, participants performed six successive trials of walking and six successive trials of running at a self-determined submaximal speed–three trials per side. A successful trial was when one leg had clean foot contact on the force platform. The intervening 5 km run occurred at a submaximal speed of 80% of their self-reported personal best speed, to simulate a casual run. The group’s mean (one standard deviation [sd]) walking speeds were 1.311 (0.10) m/s before and 1.309 (0.08) m/s after the 5 km treadmill run. The group’s mean (sd) running speeds were 3.068 (0.128) m/s before and 3.137 (0.152) m/s after the 5 km treadmill run.

### 2.3 Biomechanical processing

Motion capture data was preprocessed with a customized Matlab script, specifically, the marker trajectories and GRF were filtered at 6 Hz and 30 Hz, respectively. A threshold of 20N of the vertical ground reaction force (GRF) was used to determine initial contact (IC) and toe-off. Musculoskeletal modelling was then performed in OpenSim, using a published model (Rajagopal et al., 2016) with updated abduction-adduction and inter-external rotation in the knee joint (Mei et al., 2019). The MSK model with three degrees of freedom each (3DOFs) in hip and knee joints and 1DOF in the sagittal plane of the ankle was employed for post data processing. Inverse kinematics was used to calculate joint angles, with weighted factors to minimize the position errors between experimental markers and virtual markers. Static optimization was used to calculate individual muscle forces and muscle activations, where the muscle activation was validated against the collected EMG signals (Mei et al., 2019). JCF was then quantified by following an established pipeline of static optimization and joint reaction analysis (DeMers et al., 2014; Lerner and Browning, 2016; Mei et al., 2019).

Three trials of kinematics and contact forces from each participant were averaged to avoid the potential inter-trial variation during the walking stride and running stance. For walking, the variables were time-normalised to 101 data points between two consecutive ipsilateral ICs (i.e., stride), whilst for running, the variables were time-normalised to 51 data points between IC and toe-off (i.e., stance). JCFs were normalised to the participant’s standing bodyweight (BW) and expressed in units of BW. A Cardan XYZ rotation sequence was used to calculate 3D joint angles [26]. Positive values along the *x*-axis (medio-lateral axis) represented hip flexion, knee extension, and ankle dorsiflexion; positive values along the *y*-axis (postero-anterior axis) represented hip adduction, knee adduction; and positive values along the *z*-axis (vertical axis) represented hip and knee internal rotation. For JCFs, force along the *x*-axis represented an anterior-posterior force with positive values reflecting anterior shear, force along the *y*-axis reflecting a vertical force, with negative values reflecting compression, and force along the *z*-axis reflecting a medio-lateral force with positive values reflecting a lateral shear.

### 2.4 Machine learning

All analyses were conducted in R software (version 4.4.2) and Python (version 3.9.6), with associated codes found online (https://github.com/bernard-liew/deep-learning-on-joint-contact-forces). The code uses the *reticulate* package which provides an R interface to Python (Ushey et al., 2021), as well as the Python packages *fastai and timeseriesAI* for time-series deep learning (Howard and Gugger, 2020; Oguiza, 2022).

#### 2.4.1 Shaping input array and output matrix

Seven time-series predictors were included in the present study, which included the 3D joint angles of the hip and knee, and the ankle sagittal plane angle. The predictors were shaped into a m×n×p array, where m represents the number of observations, n the number of predictors, and p the number of cycle time points. There were nine outcomes which included the 3D JCFs of the hip, knee, and ankle joints. Each outcome was modelled separately, and was shaped into a m×p array. For both walking and running datasets, data came from 20 participants, with pre and post measurements, bilaterally, resulting in m=80 .

#### 2.4.2 Pre-processing

In order to validate our deep learning models across different gait types (walk vs. run) and also to develop a model trained on two gait types, a separate dataset “walk stance” was created. Given that the walking data represented a complete stride, the first 60% of the data, which typically defines the stance phase, was extracted and rescaled to 51 cycle points using cubic spline interpolation. No further processing was performed on the predictors and outcomes.

#### 2.4.3 Performance evaluation

Two random participants’ data was used for separate testing of the model after training has completed, one random participant’s data was used for training validation, and 17 participants’ data was used for training (Moghadam et al., 2023). Five training-testing schemes were evaluated (Table 1): 1) a model trained and validated on walking data, 2) a model trained and validated on running data, 3) a model trained on walking, and the final model was used to predict running outcomes, 4) a model training on running, to predict walking outcomes, and 5) a model training on a combined walk-run dataset, to predict walking and running outcomes.

**TABLE 1:** Deep learning models for each of nine outcomes. Each model represented the different types of gait data used for training and testing Each model was trained using six different epochs.

Models	Train set	Validation set	Test set
Train on walk, tested on walk: ${Model}_{Test=walk}^{Train=walk}$	$P_{68\;x\;7\;x\;101}^{walk}$	$P_{4\;x\;7\;x\;101}^{walk}$	$P_{8\;x\;7\;x\;101}^{walk}$
	$O_{68\;x\;101}^{walk}$	$O_{4\;x\;101}^{walk}$	$O_{8\;x\;101}^{walk}$
Train on run, test on run: ${Model}_{Test=run}^{Train=run}$	$P_{68\;x\;7\;x\;101}^{run}$	$P_{4\;x\;7\;x\;101}^{run}$	$P_{8\;x\;7\;x\;101}^{run}$
	$O_{68\;x\;101}^{run}$	$O_{4\;x\;101}^{run}$	$O_{8\;x\;101}^{run}$
Trained on run, tested on walk: ${Model}_{Test=walk}^{Train=run}$	$P_{68\;x\;7\;x\;101}^{run}$	$P_{4\;x\;7\;x\;101}^{run}$	$P_{8\;x\;7\;x\;101}^{walk}$
	$O_{68\;x\;101}^{run}$	$O_{4\;x\;101}^{run}$	$O_{8\;x\;101}^{walk}$
Trained on walk, tested on run: ${Model}_{Test=run}^{Train=walk}$	$P_{68\;x\;7\;x\;101}^{walk}$	$P_{4\;x\;7\;x\;101}^{walk}$	$P_{8\;x\;7\;x\;101}^{run}$
	$O_{68\;x\;101}^{walk}$	$O_{4\;x\;101}^{walk}$	$O_{8\;x\;101}^{run}$
Trained on combine, tested on walk: ${Model}_{Test=walk}^{Train=comb}$ And tested on run: ${Model}_{Test=run}^{Train=comb}$	$P_{136\;x\;7\;x\;101}^{comb}$	$P_{8\;x\;7\;x\;101}^{comb}$	$P_{8\;x\;7\;x\;101}^{walk}$
	$O_{136\;x\;101}^{comb}$	$O_{8\;x\;101}^{comb}$	$O_{8\;x\;101}^{walk}$
	$P_{136\;x\;7\;x\;101}^{comb}$	$P_{8\;x\;7\;x\;101}^{comb}$	$P_{8\;x\;7\;x\;101}^{run}$
	$O_{136\;x\;101}^{comb}$	$O_{8\;x\;101}^{comb}$	$O_{8\;x\;101}^{run}$

Abbreviations. P = predictor, O = outcome, comb = combined walk and run data

#### 2.4.4 Deep learning

Herein, we used the XCM architecture as proposed previously (Fauvel et al., 2021) for deep learning, given that our prior research showed that it outperformed architectures like a custom fully connected network, InceptionTime (Ismail Fawaz et al., 2020), and Time Series Transformer plus (TSTPlus) (Zerveas et al., 2021). XCM uses 2D and 1D convolutional filters, in parallel, that allows the extraction of temporal information directly from the input data (Fauvel et al., 2021), rather than from the processed features if the 2D and 1D filters were to be sequential. XCM uses 1D global average pooling to reduce the number of parameters and improve generalization ability. The model uses a rectified linear unit (ReLU) activation function for the convolutional blocks. To enable regression prediction, the final layer of this network consists of a linear layer with p units, which is represented the JCF at each p% cycle point. We trained the network using six different number of epochs (25, 50, 100, 200, 500, and 1000) all using a batch size of 34, an Adam optimiser, a learning rate of 1 × 10^−5^, a moving average coefficients β1 = 0.9, β2 = 0.99, weight decay of 0.1, and using a time-by-time root mean squared error (RMSE) loss function.

#### 2.4.5 Predictive accuracy

The prediction performance of the models was determined by comparing the nine JCFs in the test set, against their predicted values using the Root Integrated Mean Squared Error (BW), relative Root Integrated Mean Squared Error (relRMSE, %) (Ren et al., 2008), and Pearson correlation coefficient (cor) (Johnson et al., 2019a; Johnson et al., 2019b).

$$RMSE=\sqrt{\frac{\int_{0}^{T}{\left[u_{obs}\left(t\right)-u_{pred}\left(t\right)\right]}^{2}dt}{T}}$$ (1)

$$relRMSE=\frac{RMSE}{0.5\left[\sum_{i=1}^{2}\left(\max_{0<t<T}\left(u_{i}\left(t\right)\right)-\min_{0<t<T}\left(u_{i}\left(t\right)\right)\right)\right]}\;x\;100\%$$ (2)

where T represents the stance duration between initial contact and toe-off, $u_{obs}\left(t\right)$ represents the value at the $t^{th}$ time point of the observed outcome, $u_{pred}\left(t\right)$ represents the value at the $t^{th}$ time point of the predicted outcome, and i represents either the observed or predicted outcomes.

## 3 Results

**FIGURE 2:**
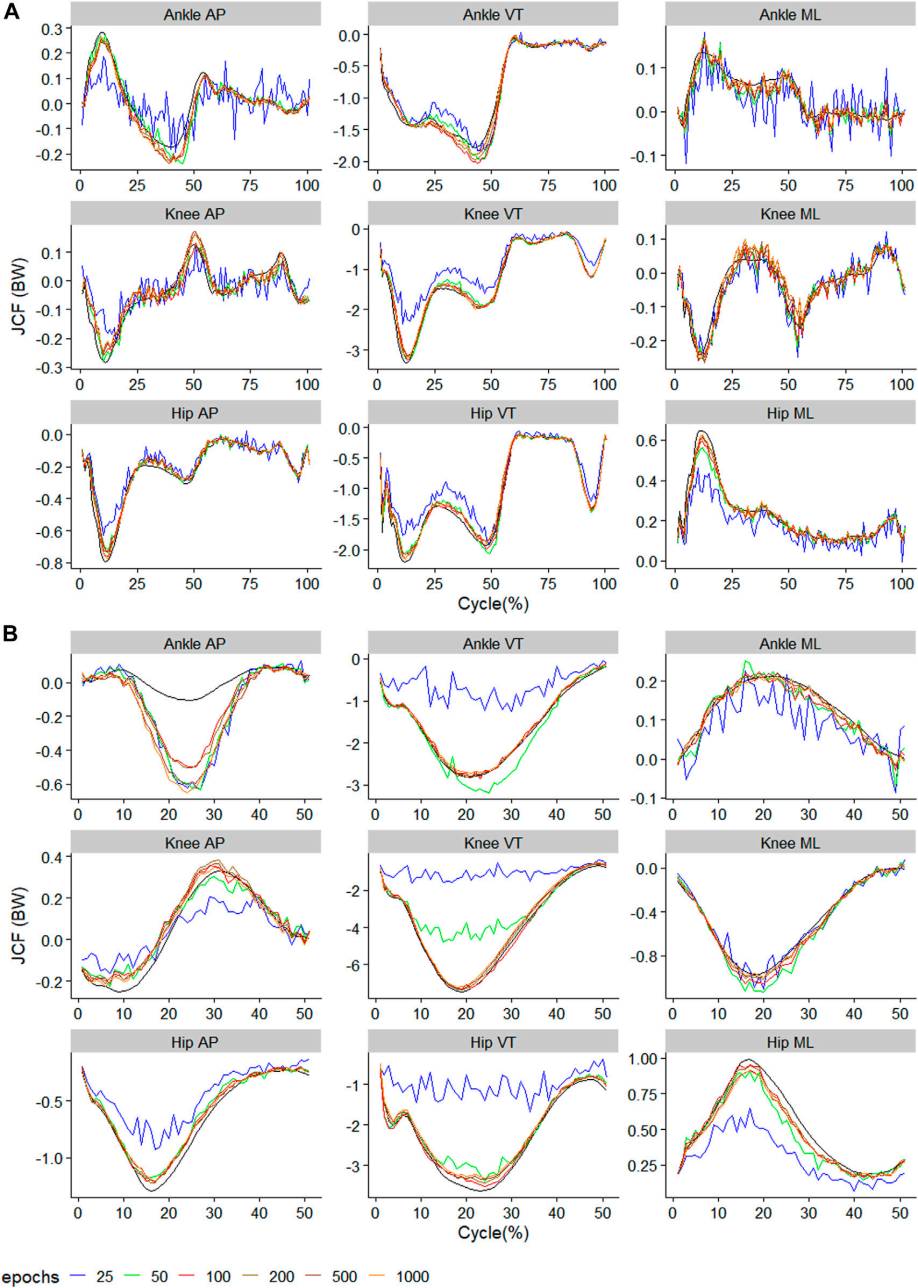
Observed (black) and predictedthree-dimensional joint contact forces of **(A)** ${Model}_{Test=walk}^{Train=walk}$ across the walking stride and **(B)** ${Model}_{Test=run}^{Train=run}$ across the running stride. The x-axis of **(A)** reflects 100 data points reflecting a walking stride, and **(B)** 50 data points reflecting a running stance. Waveforms represent the average across all test samples.

**FIGURE 3:**
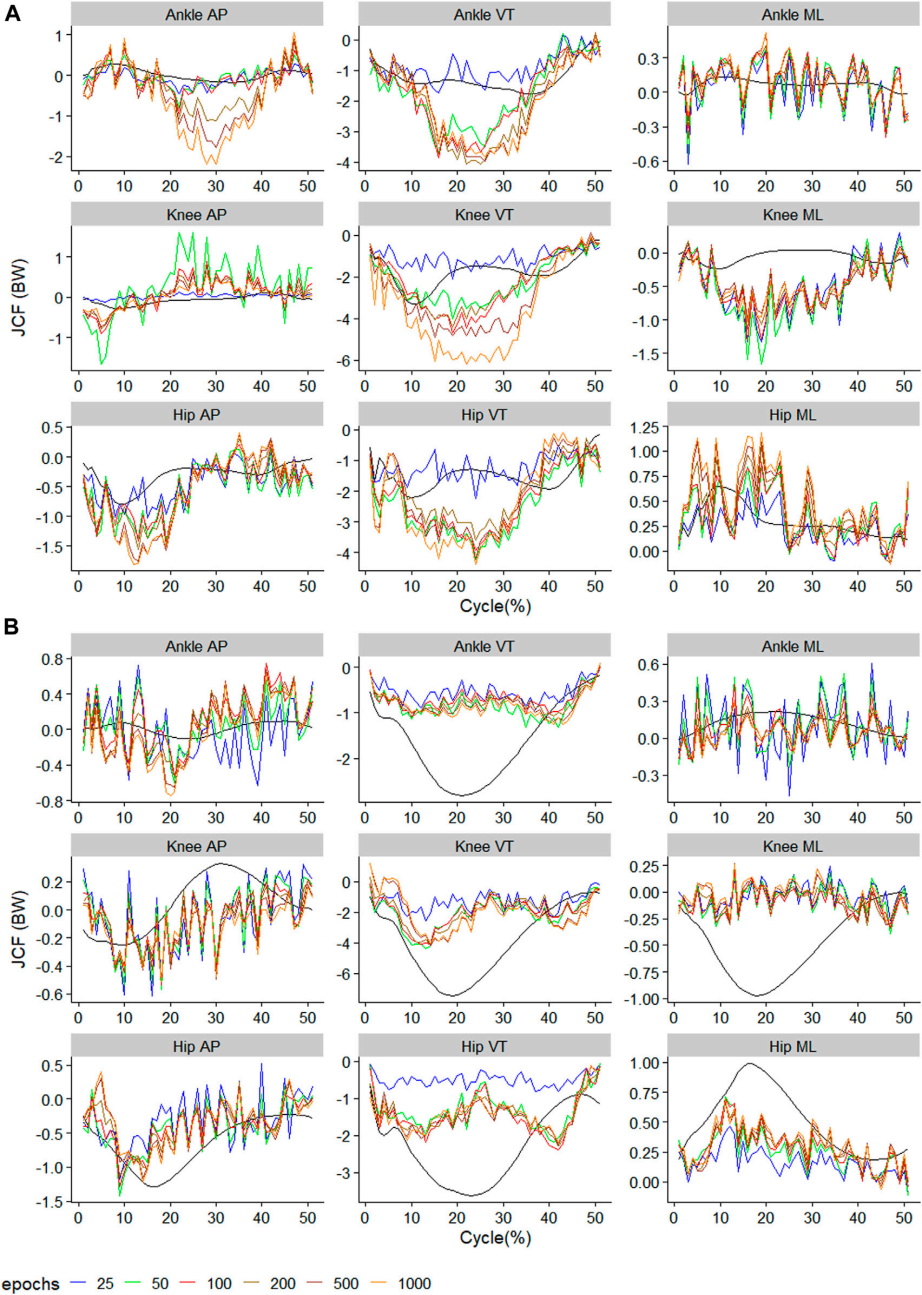
Observed (black) and predicted three-dimensional joint contact forces of **(A)** ${Model}_{Test=walk}^{Train=run}$ across the walking stride and **(B)** ${Model}_{Test=run}^{Train=walk}$ across the running stride. The x-axis of **(A)** reflects 100 data points reflecting a walking stride, and **(B)** 50 data points reflecting a running stance. Waveforms represent the average across all test samples.

**FIGURE 4:**
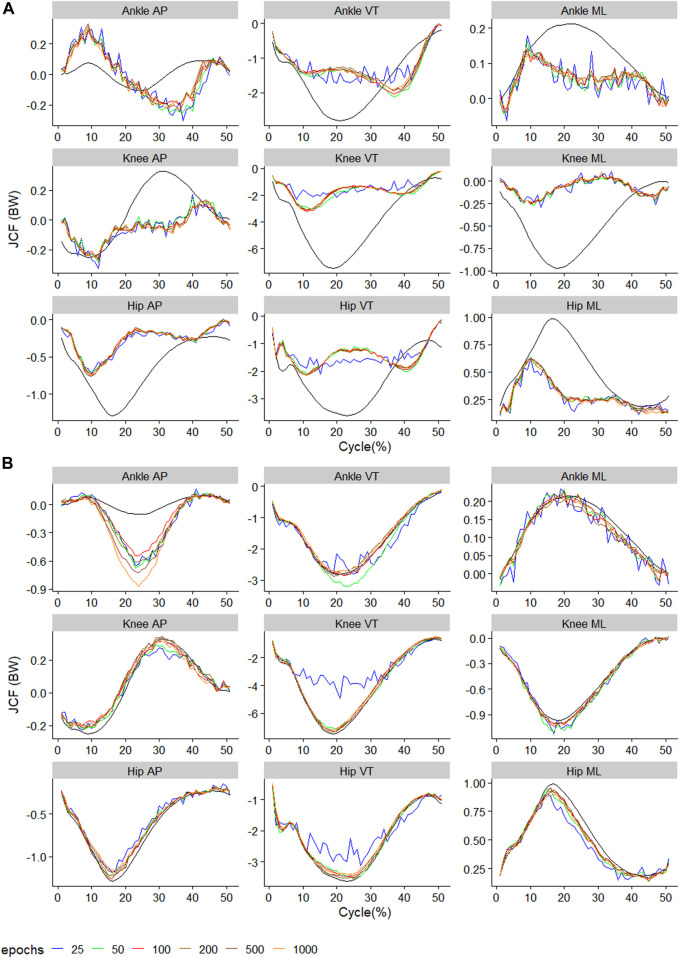
Observed (black) and predicted three-dimensional joint contact forces of **(A)** ${Model}_{Test=walk}^{Train=comb}$ across the walking stride and **(B)** ${Model}_{Test=run}^{Train=comb}$ across the running stride. The x-axis of **(A)** reflects 100 data points reflecting a walking stride, and **(B)** 50 data points reflecting a running stance. Waveforms represent the average across all test samples.

The raw waveform of the predictors and outcomes used in the present study can be found in the Supplementary Material. The performance metrics of the best model for each outcome can also be found in the Supplementary Material. The observed and predicted mean waveform for each of the nine outcomes are presented in Figures 2–4. A general observation is that training using too few epochs (<100) results in predicted JCF waveforms with high “wiggleliness”.

### 3.1 Training and testing on the same gait

**FIGURE 5:**
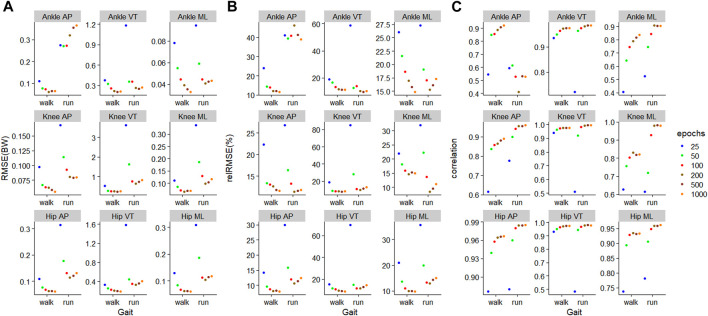
Prediction performances of machine learning models involving different training epochs and gait types, using the models ${Model}_{Test=walk}^{Train=walk}$ for walking and ${Model}_{Test=run}^{Train=run}$ for running. **(A)** Root mean squared error, **(B)** relative root mean squared error, and **(C)** correlation.

For walking, 1000 epochs resulted in the lowest RMSE in five out of nine outcomes, whilst for running, 200 epochs resulted in the lowest RMSE in six outcomes (Figure 5). For walking, in all nine outcomes, the biggest improvement in RMSE occurred when increasing the number of training epochs from 25 to 50 (Figure 5). For running, for eight outcomes, the biggest improvement in RMSE occurred when increasing the number of training epochs from 25 to 50 (Figure 5).

The outcome with the smallest RMSE was ankle medial-lateral JCF with values of 0.03BW and 0.04BW for walking and running, respectively (Figure 5). In contrast, the outcome with the biggest RMSE was the knee VT JCF with values of 0.24BW and 0.65BW for walking and running, respectively (Figure 5). In general, JCF in the medial-lateral plane resulted in the lowest average RMSE of 0.07 (0.02) BW and 0.12 (0.08) BW for walking and running, respectively (Figure 5). JCF in the VT plane resulted in the greatest RMSE of 0.27 (0.08) BW and 0.80 (0.83) BW for walking and running, respectively (Figure 5). When comparing the relRMSE, the average performance in the prediction outcomes of different axes in walking ranged from 11.6% to 16.1%, whilst that of running ranged from 17.8% to 24.0% (Figure 5).

### 3.2 Training and testing on different gait

**FIGURE 6:**
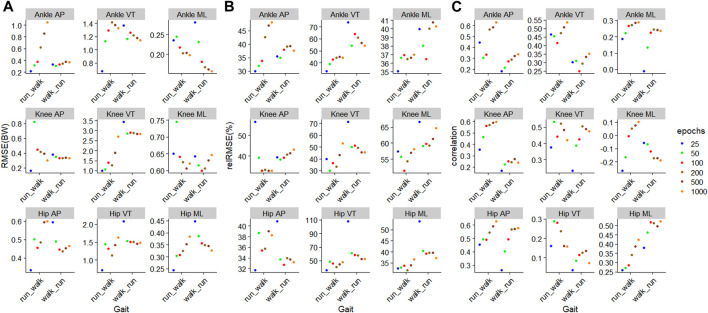
Prediction performances of machine learning models involving different training epochs and gait types, using the models ${Model}_{Test=walk}^{Train=run}$ for walking and ${Model}_{Test=run}^{Train=walk}$ for running. **(A)** Root mean squared error, **(B)** relative root mean squared error, and **(C)** correlation.

During the ${Model}_{Test=walk}^{Train=run}$ , training using 25 epochs resulted in the lowest RMSE on 7 out of 9 outcomes, while for the ${Model}_{Test=run}^{Train=walk}$ , training using 1000 epochs resulted in the lowest RMSE on 4 outcomes (Figure 6). For ${Model}_{Test=walk}^{Train=run}$ , the biggest improvement in RMSE occurred with moving from 50–100 epochs (4 outcomes), and moving from 500–1000 epochs (4 outcomes), while for ${Model}_{Test=run}^{Train=walk}$ , the biggest improvement occurred from increase training from 25 to 50 epochs in eight outcomes (Figure 6). The outcome with the smallest RMSE was the knee AP JCF with a value of 0.16BW for ${Model}_{Test=walk}^{Train=run}$ , and the ankle medial-lateral JCF with a value of 0.16BW for ${Model}_{Test=run}^{Train=walk}$ (Figure 6). The outcome with the biggest RMSE was the knee VT JCF for ${Model}_{Test=walk}^{Train=run}$ at 1.00BW and for ${Model}_{Test=run}^{Train=walk}$ at 2.84BW (Figure 6).

### 3.3 Training on both gait types

**FIGURE 7:**
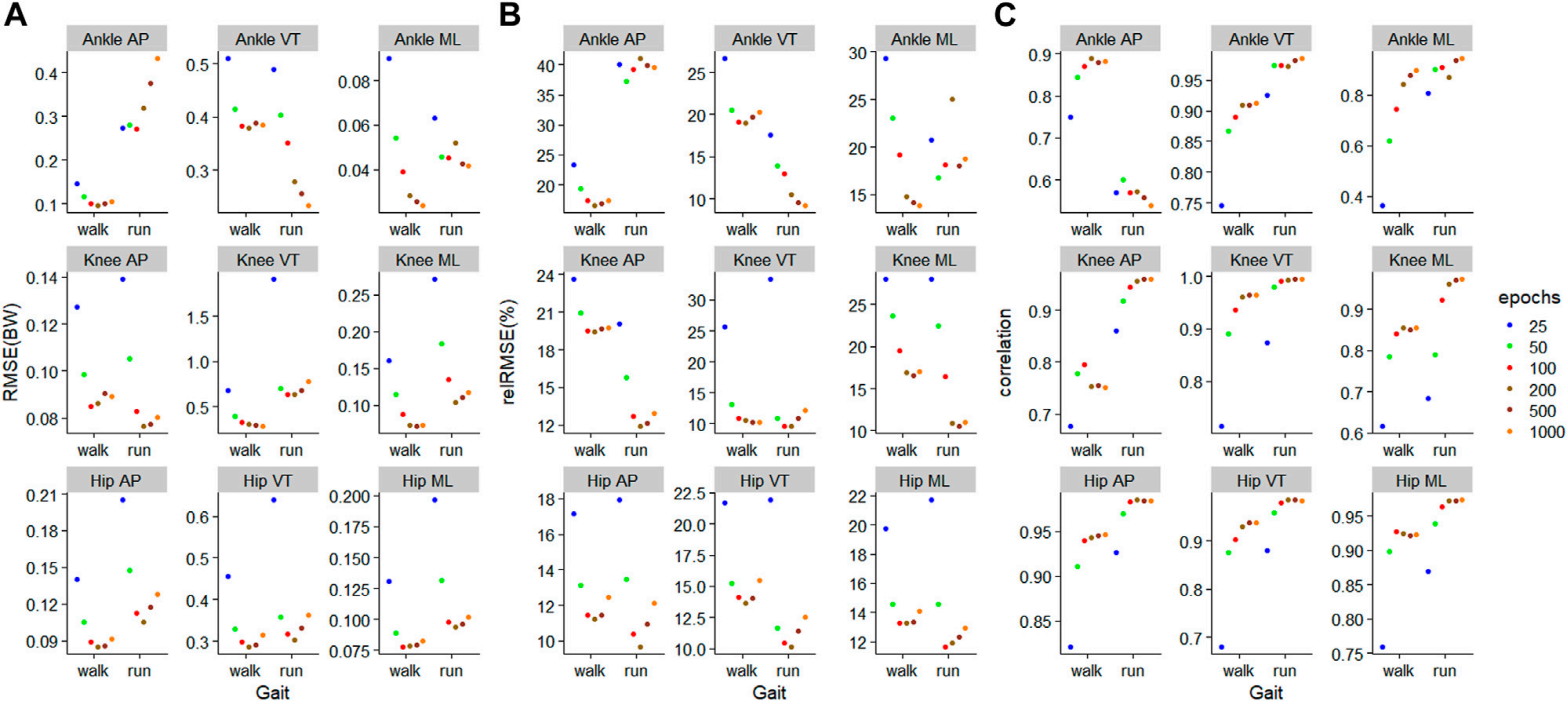
Prediction performances of machine learning models involving different training epochs and gait types, using the models ${Model}_{Test=walk}^{Train=comb}$ for walking and ${Model}_{Test=run}^{Train=comb}$ for running. **(A)** Root mean squared error, **(B)** relative root mean squared error, and **(C)** correlation.

For the ${Model}_{Test=walk}^{Train=comb}$ , training using 200 epochs resulted in the lowest RMSE on four outcomes (Figure 7). For the ${Model}_{Test=run}^{Train=comb}$ , training using 200 epochs resulted in the lowest RMSE on six outcomes (Figure 7). The biggest improvement in RMSE occurred with moving from 25–50 epochs across all outcomes in ${Model}_{Test=walk}^{Train=comb}$ , and in eight outcomes for ${Model}_{Test=run}^{Train=comb}$ . The outcome with the smallest RMSE was ankle medial-lateral JCF with values of 0.02BW for ${Model}_{Test=walk}^{Train=comb}$ and 0.04BW for ${Model}_{Test=run}^{Train=comb}$ (Figure 7). The outcomes with the biggest RMSE were the ankle VT for ${Model}_{Test=walk}^{Train=comb}$ at 0.38BW, and knee VT for ${Model}_{Test=run}^{Train=comb}$ at 0.63BW (Figure 7). When comparing the relRMSE, the average performance in the prediction outcomes of different axes in walking ranged from 16.7% to 18.0%, whilst that of running ranged from 13.2% to 22.1% (Figure 7).

## 4 Discussion

This is the first study to the authors’ knowledge to predict JCFs across all three major lower-limb joints in walking and running using motion capture-based kinematics. Our findings supported the first hypothesis in that our ML models could predict JCFs within the range of MDC values reported for JCFs. A previous reported study reported that the knee JCF MDC values were between 0.43 and 1.53 bodyweight (BW) (Price et al., 2017). However, these performances were not replicated when training and testing were performed on different gait types. The second hypothesis was not supported in that training up to 1000 epochs did improve the ML performance in some instances, but degraded performance in other instances. In contrast to the third hypothesis, ML models trained and tested on different gait types had clinically significantly worse performance (four times worse) than models trained and tested on identical gait types. Lastly, combining both walking and running gait data did not appreciably alter ML performance compared to ML models trained and tested on a single gait type.

Previous ML studies predicting the knee JCF have reported a correlation between the observed and predicted forces between 0.85 and 0.94, and a Normalised RMSE (NRMSE) (%) between 4.5% and 13.3% (Ardestani et al., 2014; Rane et al., 2019; Giarmatzis et al., 2020). For our knee JCFs, our average correlation magnitude was 0.87, and relative RMSE was 15.4%. A notary caution when comparing studies is the differences in error metrics used. Presently, the calculated relative RMSE reflects the integral of the errors across the gait cycle investigated, but the NRMSE used in previous studies reflected the average RMSE across the gait cycle (Ardestani et al., 2014; Rane et al., 2019; Giarmatzis et al., 2020). The much better predictive performance of Giarmatzis et al. (Giarmatzis et al., 2020) compared to the present study, could be due to the large number of test trials available for model training (n = 54, number of observations = 4784), and the inclusion of GRFs as well as joint angles for prediction.

When considering the relative RMSE of the ML models on JCFs presently, the performance was slightly worse than a previous study on running on joint moments (Liew et al., 2021). The best relative RMSE values of the vertical JCFs in running of the ankle (11.5%), knee (10.4%), and hip (11.4%) in the present study, but the same metric values of the sagittal plane joint moments of the ankle, knee, and hip, were 5%, 7%, and 12%, respectively (Liew et al., 2021). However, a previous study included 490 samples in their training dataset with 27 joint kinematics as predictors (Liew et al., 2021). Johnson et al. (Johnson et al., 2019b) reported a relRMSE in the 3D knee joint moments of 13.8%–31.8% in walking and 7.8%–31.7% in running, with 570–646 and 233–884 samples in the combined training and testing datasets. Interestingly, the same relative prediction performance was achieved in walking, as in running, even though the biomechanical variables were “noisier” in running compared to walking, due to established issues like soft-tissue artefacts. For example, the best relative RMSE values of the vertical JCFs in walking of the ankle (15.5%), knee (8.1%), and hip (8.9%) were similar to that of running in the present study.

Conventional ML wisdom of the *Bias-Variance* trade-off predicts that ML performance on a separate test set will degrade after an optimal number of training epochs is reached. However, a general pattern can be observed that the greater the number of training epochs, ML prediction performance in general improved. In addition, the predicted waveforms became “smoother” and more comparable to the original JCFs. One study predicting joint moment waveforms trained all ML models using 200 epochs (Liew et al., 2021), whilst another used 1000 epochs (Wang et al., 2020), and some others not reporting this hyperparameter (Johnson et al., 2019b). The present study findings suggest that the results of prior studies could have been improved if a different number of training epochs were to be used. It is challenging to suggest an optimal training epoch that is generalisable to all ML situations. However, the present study suggests that if the goal is to achieve high-performance “smooth” waveform predictions, training epochs should generally be >100.

It is interesting to speculate on the statistical mechanisms for why a low number of training epochs results in “wiggleliness” of the waveforms. For irregular data (functions not observed at equidistant time points), integration weights are required to weight the different RMSE values differently, which could influence the “wiggleliness”. However, even for regularly spaced time-series data “wiggleliness” can happen. This could be due to a lack of a missing smoothness penalty in the loss function during training. Interestingly, increasing the training epochs appeared to result in the network “learning” the smoothness of the outcomes. This present finding could be attributed to the double descent phenomenon (Nakkiran et al., 2021). This phenomenon predicts that with a greater number of training epochs, the learned relationship on the training data achieves near perfect fit, resulting in an interpolation through the data (Belkin et al., 2019). It may be that for prediction problems where smoothness in the prediction of outcomes are desired, early stopping for ML algorithms may not be optimal (Belkin et al., 2019). It is interesting to note also that ReLU networks, such as XCM, can approximate smooth functions of any order (Zhang and Wang, 2022) and are essentially spline interpolators (Savarese et al., 2019). Previous work has suggested that the number of layers with ReLU activation units could influence the smoothness of predictions (Savarese et al., 2019). Whilst both the activation function and number of layers were constant across all models presently investigated, future studies that investigate the effects of these architectural parameters on the smoothness of prediction is essential.

The poor ML performance when training and testing on data from different gait types suggest the statistical relationship learned by ML models may not fully approximate physical laws sufficiently, to allow for cross-gait prediction application. One reason for this could be that the values of biomechanical variables between different gait types have different peak values, different timing of the peak values, and different spread of values. The ML model algorithm used presently is modelling the conditional distribution of the mean values of the outcome given a set of predictors, which neglects other aspects of the outcome, such as its spread (e.g., variance, kurtosis). Increasingly, deep learning algorithms that can simultaneously model the conditional distribution of both the mean and spread of the outcome are being developed (Kook et al., 2022), although their potential impact on cross-gait prediction has not been explored.

Our experimental design precludes us from disentangling the effects of different gait types and the effects of speed, as the cause of the poor ML performance. Speculatively, we hypothesise that the poorer cross-gait predictive performance is primarily caused by the different biomechanical requirements of distinct gait types (Schache et al., 2015), rather than speed effects. This is because distinct biomechanical characteristics are observed when walking and running at the same speed, such as a much greater hip power contribution in the former than the latter (Schache et al., 2015). Also, the shape of kinematic and kinetic waveforms has greater variation between gait types, than between speed variations (Schache et al., 2015). Future studies investigating the limits in generalisability of the ML performance across different distinct gait types and variants within gait types (e.g., different speeds) should be explored.

This study is not without limitations. First, ideally, ML models to predict JCFs should be developed using direct *in-vivo* measurements collected *via* instrumented implants (Bergmann et al., 1993; Fregly et al., 2012). However, direct *in-vivo* measurements are very challenging to collect and are often performed on patients with orthopaedic disorders, where the performance of more strenuous physical activities is not possible (Bergmann et al., 1993; Fregly et al., 2012). This explains why the number of participants where direct *in-vivo* data are available is very low (e.g., n = 2 in the Knee Grand Challenge). Given that ML requires much more data than musculoskeletal modelling, training a ML model using current publicly available direct *in-vivo* data is not possible. Developing ML models to predict JCFs based on musculoskeletal models represents the most feasible way at present, to fully realising the potential of bringing biomechanical measurements from the lab into clinical environments. Second, there are many optimisation methods available to calculate the muscle forces needed to quantify JCFs (Trinler et al., 2018). The accuracy of our ML model in estimating JCFs will only be as accurate as the accuracy of the initial musculoskeletal modelling approach in quantifying JCFs, and the latter should be based on the intended application of the ML model.

Third, we used predictors derived from motion capture cameras which although portable, are not ubiquitously available in the clinic and the field. Wearable sensors, such as accelerometers, represent the most clinically feasible methods of measuring body motion. However, wearable sensor signals may be “nosier” than kinematics collected from optical cameras. For example, one study which used IMU variables as input resulted in a RMSE knee extensor moment of 1.13 Nm/kg (Stetter et al., 2020), whilst another study using optical camera inputs had a RMSE of 0.25 Nm/kg (Liew et al., 2021) during running. Whether the performance of ML modelling to estimate JCFs using these alternative motion technologies would match that of traditional motion capture camera needs to be investigated. Lastly, the present study used biomechanical features that have been averaged across trials and time-normalised. These processing steps may result in over optimistic ML performance, given that “noise” to the signals are reduced. The extent “noise” should be removed by signal pre-processing in ML studies should be based on the intended use case of the ML model. If the ML model is intended for real-time streaming of step-by-step JCFs, then ML models should be trained on the original signals (Wang et al., 2020; Camargo et al., 2022). However, if the ML model is intended for “post-hoc” prediction of the average gait cycle’s JCFs, then our approach may be suitable.

## 5 Conclusion

ML can be used to predict JCFs of the lower limb during walking and running, to a degree that is within the current MDC values of JCFs. When using deep learning models, like in the present study, training using too few epochs (<100) generally leads to not only poor prediction performances but excessive “wiggleliness” of the waveforms. ML models trained on one gait type cannot be applied to another gait type. If ML models are required for cross-activity usage, that training needs to be done on data from all intended activities.

## Data Availability

The datasets presented in this study can be found in online repositories. The names of the repository/repositories and accession number(s) can be found below: https://auckland.figshare.com/projects/Dataset_of_Lower_Extremity_Joint_Angles_Moments_and_Forces_in_Distance_Running/136708.
