# Supplementary material for: Smooth and accurate predictions of joint contact force time-series in gait using over parameterised deep neural networks
Source: Front Bioeng Biotechnol. 2023 Jul 3;11:1208711. doi: 10.3389/fbioe.2023.1208711 (PMC10350628; doi:10.3389/fbioe.2023.1208711)
Supplement: Supplementary file 1 [file DataSheet1.docx]

**Supplementary material**

Smooth and accurate predictions of joint contact force time-series in gait using over parameterised deep neural networks

Bernard X.W. Liew ^1^, David Rügamer ^2,3^, Qichang Mei ^4,5,6^, Zainab Altai^1^, Xuqi Zhu^7^, Xiaojun Zhai^7^, Nelson Cortes^1,8^

^1^School of Sport, Rehabilitation and Exercise Sciences, University of Essex, Colchester, Essex, United Kingdom

^2^Department of Statistics, Ludwig-Maximilians-Universität München, Munich, Germany

^3^Munich Center for Machine Learning, Munich, Germany

^4^Faculty of Sports Science, Ningbo University, Ningbo, China

^5^Research Academy of Grand Health, Ningbo University, Ningbo, China

^6^Auckland Bioengineering Institute, The University of Auckland, Auckland, New Zealand

^7^School of Computer Science and Electrical Engineering, University of Essex, Colchester, Essex, United Kingdom

^8^Department of Bioengineering, George Mason University, Fairfax VA, USA

**Journal Category:** Original article

**Address for reprint requests and other correspondence:** Bernard Liew, School of Sport, Rehabilitation and Exercise Sciences, University of Essex, Colchester, Essex, United Kingdom; E-mail: [bl19622@essex.ac.uk](mailto:bl19622@essex.ac.uk); [liew_xwb@hotmail.com](mailto:liew_xwb@hotmail.com). Tel: +44 120 687 3522


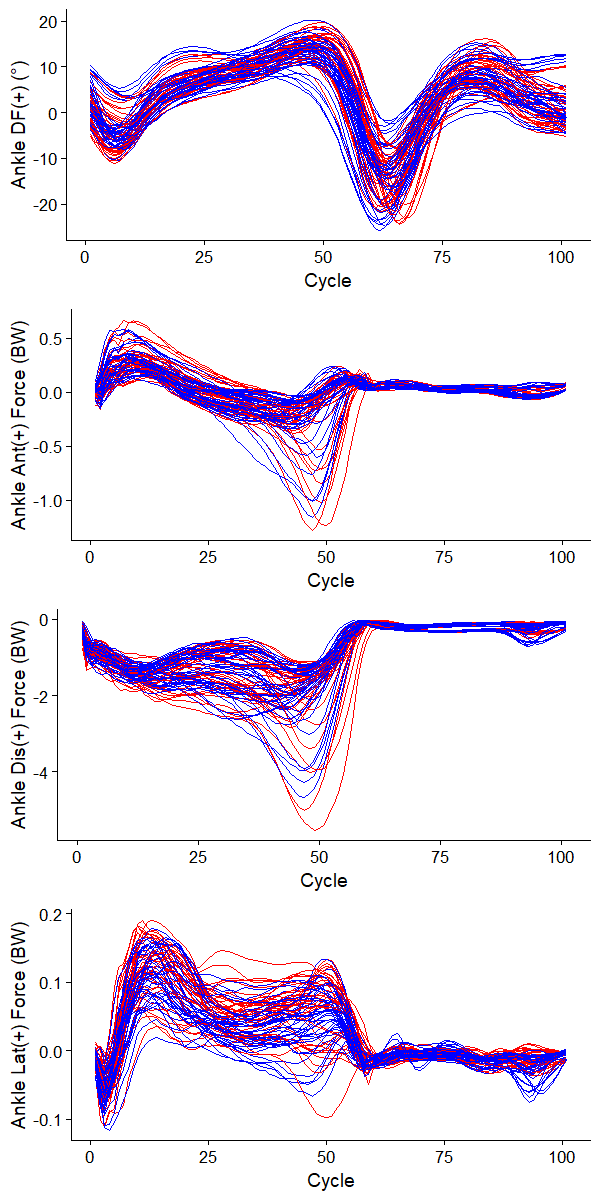


Figure 1. Individual waveforms of ankle angle and contact forces across the walk stride cycle (%).


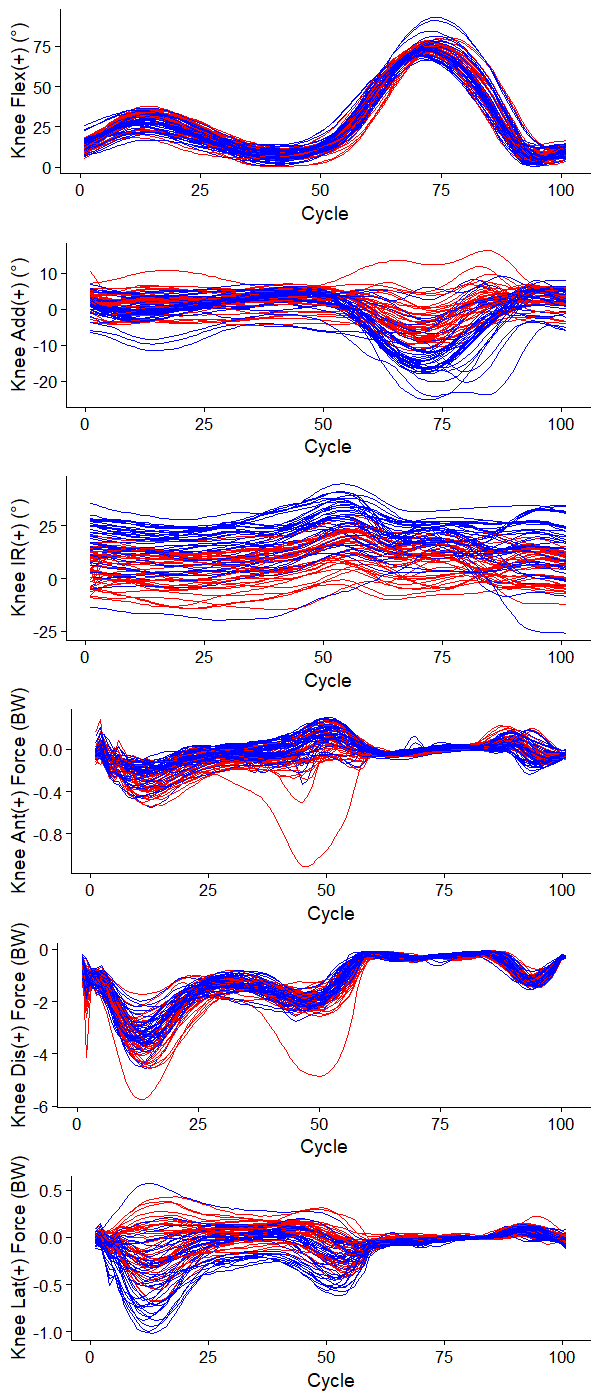


Figure 2 Individual waveforms of knee angle and contact forces across the walk stride cycle (%).


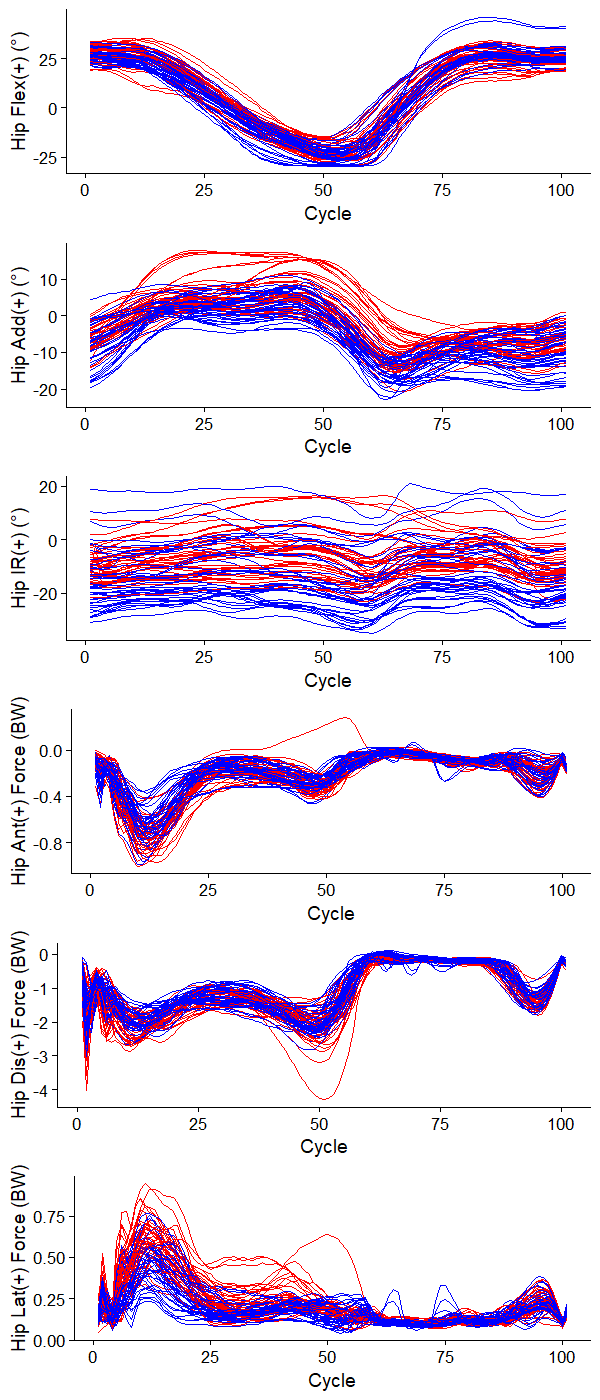


Figure 3 Individual waveforms of hip angle and contact forces across the walk stride cycle (%).


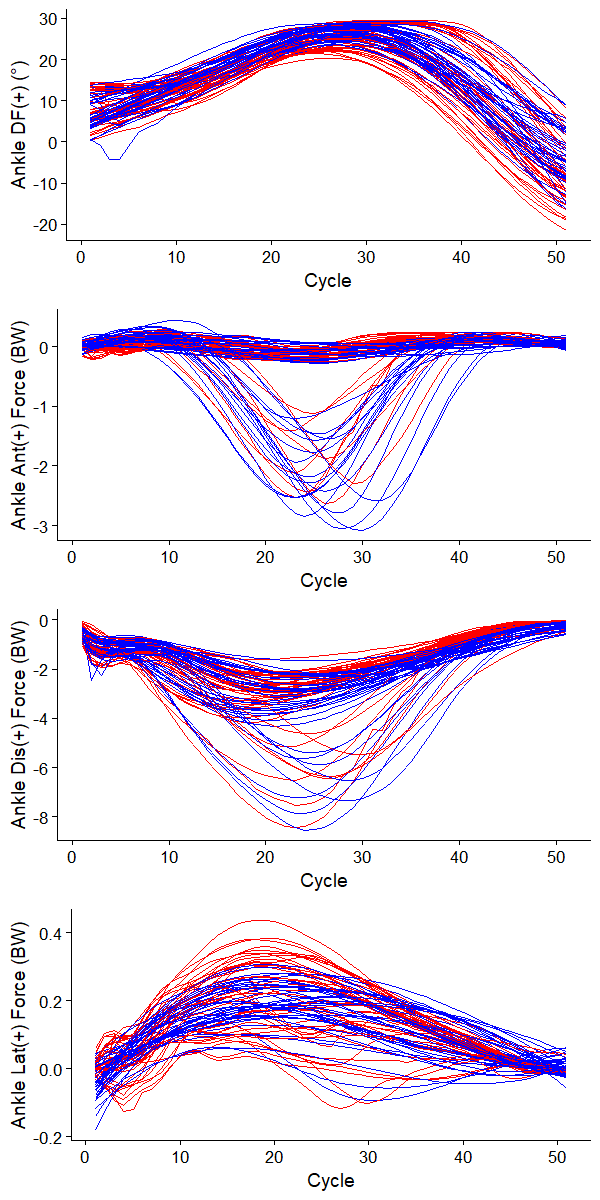


Figure 4 Individual waveforms of ankle angle and contact forces across the run stance cycle (%).


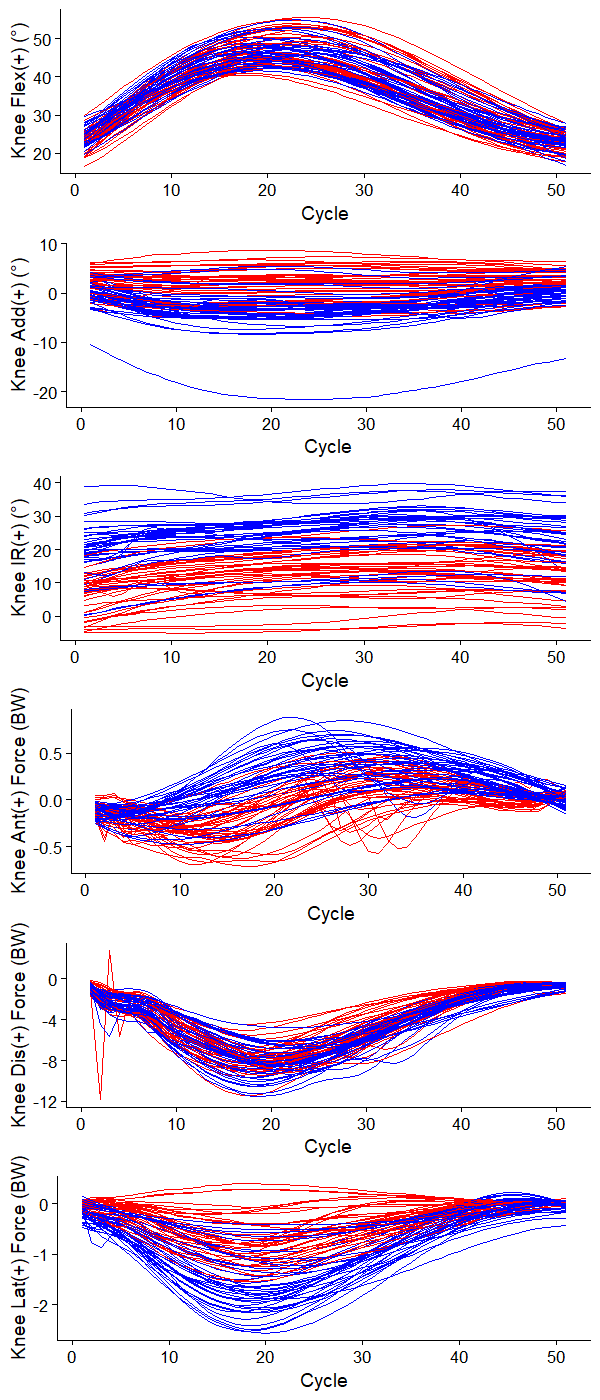


Figure 5 Individual waveforms of knee angle and contact forces across the run stance cycle (%).


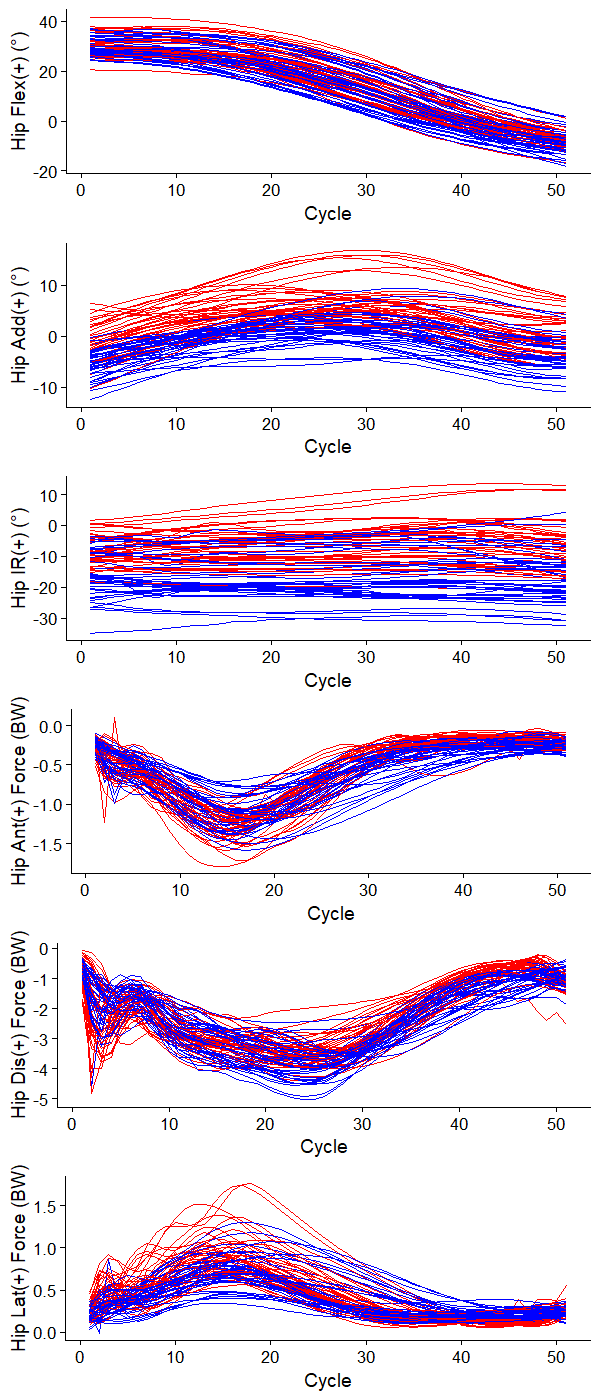


Figure 6 Individual waveforms of hip angle and contact forces across the run stance cycle (%).

Table 1. Error metrics for each outcome for the best model, trained and tested on the same gait type

| **outcomes** | **type** | **Best RMSE** | **Best reRMSE** | **Best correlation** |
| --- | --- | --- | --- | --- |
| Ankle AP | walk | 0.06 | 11.66 | 0.92 |
| Ankle VT | walk | 0.21 | 11.29 | 0.97 |
| Ankle ML | walk | 0.03 | 14.9 | 0.84 |
| Knee AP | walk | 0.06 | 11.58 | 0.89 |
| Knee VT | walk | 0.24 | 8.04 | 0.98 |
| Knee ML | walk | 0.07 | 14.61 | 0.83 |
| Hip AP | walk | 0.06 | 8.04 | 0.97 |
| Hip VT | walk | 0.19 | 8.78 | 0.97 |
| Hip ML | walk | 0.06 | 9.88 | 0.94 |
| Ankle AP | run | 0.27 | 39.05 | 0.61 |
| Ankle VT | run | 0.25 | 9.8 | 0.99 |
| Ankle ML | run | 0.04 | 15.26 | 0.91 |
| Knee AP | run | 0.08 | 11.42 | 0.96 |
| Knee VT | run | 0.65 | 9.89 | 0.99 |
| Knee ML | run | 0.1 | 8.48 | 0.98 |
| Hip AP | run | 0.11 | 10.61 | 0.98 |
| Hip VT | run | 0.33 | 11.34 | 0.98 |
| Hip ML | run | 0.1 | 12.88 | 0.96 |
| **Abbreviations:** AP – anterior posterior, ML – medial-lateral, VT – vertical, RMSE – root mean squared error, relRMSE - relative root mean squared error | | | | |

Table 2. Error metrics for each outcome for the best model, trained and tested on the different gait type

| **outcomes** | **type** | **Best RMSE** | **Best reRMSE** | **Best correlation** |
| --- | --- | --- | --- | --- |
| Ankle AP | run_walk | 0.22 | 29.95 | 0.63 |
| Ankle VT | run_walk | 0.68 | 32.99 | 0.54 |
| Ankle ML | run_walk | 0.2 | 35.08 | 0.29 |
| Knee AP | run_walk | 0.16 | 32.95 | 0.6 |
| Knee VT | run_walk | 1 | 30 | 0.53 |
| Knee ML | run_walk | 0.61 | 51.38 | 0.1 |
| Hip AP | run_walk | 0.33 | 31.68 | 0.63 |
| Hip VT | run_walk | 0.72 | 36.02 | 0.29 |
| Hip ML | run_walk | 0.24 | 31.7 | 0.42 |
| Ankle AP | walk_run | 0.31 | 34.94 | 0.34 |
| Ankle VT | walk_run | 1.14 | 54.31 | 0.35 |
| Ankle ML | walk_run | 0.16 | 36.49 | 0.24 |
| Knee AP | walk_run | 0.33 | 38.3 | 0.27 |
| Knee VT | walk_run | 2.84 | 45.45 | 0.51 |
| Knee ML | walk_run | 0.6 | 59.06 | -0.06 |
| Hip AP | walk_run | 0.44 | 32.72 | 0.58 |
| Hip VT | walk_run | 1.46 | 52.37 | 0.13 |
| Hip ML | walk_run | 0.33 | 37.15 | 0.52 |
| **Abbreviations:** AP – anterior posterior, ML – medial-lateral, VT – vertical, RMSE – root mean squared error, relRMSE - relative root mean squared error | | | | |

Table 3. Error metrics for each outcome for the best model, trained and tested on combined gait type

| **outcomes** | **type** | **Best RMSE** | **Best reRMSE** | **Best correlation** |
| --- | --- | --- | --- | --- |
| Ankle AP | walk | 0.1 | 16.6 | 0.89 |
| Ankle VT | walk | 0.38 | 18.94 | 0.91 |
| Ankle ML | walk | 0.02 | 13.9 | 0.9 |
| Knee AP | walk | 0.08 | 19.44 | 0.79 |
| Knee VT | walk | 0.28 | 10.21 | 0.96 |
| Knee ML | walk | 0.07 | 16.52 | 0.85 |
| Hip AP | walk | 0.08 | 11.21 | 0.95 |
| Hip VT | walk | 0.29 | 13.64 | 0.94 |
| Hip ML | walk | 0.08 | 13.26 | 0.93 |
| Ankle AP | run | 0.27 | 37.32 | 0.6 |
| Ankle VT | run | 0.23 | 9.23 | 0.99 |
| Ankle ML | run | 0.04 | 16.75 | 0.94 |
| Knee AP | run | 0.08 | 11.94 | 0.96 |
| Knee VT | run | 0.63 | 9.64 | 0.99 |
| Knee ML | run | 0.1 | 10.56 | 0.97 |
| Hip AP | run | 0.1 | 9.68 | 0.99 |
| Hip VT | run | 0.3 | 10.16 | 0.98 |
| Hip ML | run | 0.09 | 11.67 | 0.97 |
| **Abbreviations:** AP – anterior posterior, ML – medial-lateral, VT – vertical, RMSE – root mean squared error, relRMSE - relative root mean squared error | | | | |
